# Supplementary material for: The citizen’s perception of a shared responsibility during the COVID-19 management: Insights from a focus group study across four European countries
Source: PLoS One. 2025 May 27;20(5):e0322019. doi: 10.1371/journal.pone.0322019 (PMC12112160; doi:10.1371/journal.pone.0322019)
Supplement: S1 Annex — (PDF) [file pone.0322019.s001.pdf]

PROTOCOL FOCUS GROUP DISCUSSION

# The citizen's perception of its role in the COVID-19 preparedness and response

Contact: Sandra Kamga (sandra.kengne.kamga.mobou@rivm.nl)

SHARP VERSION ENG November 30th 2020

# Introduction

The focus group discussion will take place as part of a research study lead by PhD student Sandra Kamga, as part of the EU Joint Action SHARP. During this study researchers will investigate European Union citizens' experiences, thoughts and opinions regarding their role during the preparation for, and response to, the COVID-19 pandemic.

## The background of an online meeting

A focus group discussion is an appropriate method to gather the opinions and experiences of specific groups regarding a specific topic. A Constructivitionist approach is used during a focus group, within which it is agreed that there is no absolute truth and that the reality described during a focus group discussion is the reality as the participants experience is. The informal context of a focus group discussion can encourage participants to share their opinions and experiences. The aim is not for the participants to reach consensus but for them to exchange thoughts. The advantages of a focus group include the fact data is collected for multiple participants at once and that information can be gathered on three levels, namely the individual level, the group level and the interaction.

## The goals of the focus group discussion

During the focus group data will be gathered to answer the following research question:

1. What is the citizen's perception of its role during preparation for, and response to, the COVID-19 pandemic?

## The focus group discussion

There will be a total of four digital focus groups of maximum 9 persons (n=36) per participating country. Considering age has played an important role in risk communication during this crisis, the focus groups will contain participants of similar ages. There will be the following age categories: 18-30, 31-45, 46-65, and 66 years and older.

A moderator and an assistant will lead the focus group discussions. They will ensure that the scope of the focus group discussions is clear, facilitate the discussion when necessary, and take note of the participants' comments and behaviour. The focus group discussion should not be a question-and-answer session the participant should will be given ample opportunity to talk amongst themselves (see appendix 1 for the moderator and assistant's task).

**Respondents**

## Participants

Each participating EU country will aim to include approximately 27 participants who are willing to participate in one of the focus groups. The aim is to have diversity in terms of gender and educational level. This is to ensure that a broad spectrum of opinions (and argumentation) is collected.

Sampling

- Every country will describe and execute a sampling strategy most feasible and appropriate in their country.
- The aim is to have an as representative sample of the population as possible.

For example, the Netherlands will use 1000 addresses provided in our national postal service's sample package of (to our knowledge) randomly selected addresses

## Facilities

- The focus group will take place online.

## Language

The focus groups will take place in the country's national language. If the country has more than one national languages, the language the highest percentage of the population speaks will be chosen.

The moderators from the different participating countries will receive an English version of the protocol. They will be asked to translate the protocol to the language they intend to conduct the focus groups. The translated protocols will be back translated to English by another independent individual. This will allow for changes in meaning due to translation to be identified and the translation to be adjusted as necessary. This is to ensure consistency across all participating countries.

# Protocol online focus group

This is a step-by-step guide for conducting of the focus group discussions. It is important that the key questions are asked and key messages are conveyed, yet the moderator has the freedom to conduct the focus groups as (s)he wishes. The moderator must take the national context, the age category and the general atmosphere within the focus groups into consideration.

## WELCOME AND INTRODUCTION (10 MINUTES)

**Moderator:** Good morning/ good afternoon, everyone. Thank you for attending this focus group discussion. I would like to start by introducing myself…. I come from and … and live in …I am a…. [profession]. I am working in a team researching public health emergencies. This is the reason why I have invited you today as I am interested in your experiences, opinions and ideas about the role of the citizen such as you and I in the preparation for and response to public health emergencies, such as the COVID-19. Today I am here with my colleague...

[**Assistant introduces him/herself**].

We are excited that you are here today, and we are interested in what you have to say. I would like to ask everyone to turn on their camera. I now give you the opportunity to introduce yourselves. I have a list of names here, X would you like to start. Please state your name, where you live and what your hobbies are. If you like you could share your profession.

**[The participants introduce themselves]**

I am happy to meet you all! As I mentioned earlier, I am particularly interested in your experiences, thoughts and opinions on the citizen's role of the before and during the COVID-19 pandemic. I am interested in what all of you have to say. There are no right or wrong answers and you do not necessarily have to agree with each other. There is a plenty of room for dialogue and discussions amongst yourselves. Feel free to express your own opinion.

I am the moderator and my primary task is to guide the discussion and to ensure the discussion stays within the scope of this current research study. You will do most of the talking but I will ask a couple of questions and summarize what has been said, when appropriate.

I would like to record this discussion for data analysis later. After transcribing this session, we will anonymise all input so that statements cannot be linked to a specific individual. Is there anyone who has not received an email about this? Is there anyone who has any objections to me recording this session? If you have an objection to this, I kindly ask you to disconnect from this focus group discussion. For those who do not have an objection, I will now start recording.

**[Start recording]**

To confirm on tape that you have no objections to me recording, I will now call out your names one by one and you can simply respond with 'no objection'.

This focus group discussion will last approximately 2 hours with a break half way through. At the end of this discussion you will receive a gift card per mail as a token of our appreciation.
Are there any questions? Is everything clear?

Just a couple of instructions to facilitate this digital focus group:
- Please turn on your camera
- Please mute your microphone when you are not speaking
- Raise your hand or send a message in chat box if you want to say something. I will call on you

## QUESTION 1 (10 MINUTES)

Aim: To gather information on the degree to which the participants are familiar with the COVID-19 pandemic and what their sources of information are. These answers also help contextualise the following responses.

**The moderator says:**
*What do you know about the COVID-19 pandemic?*

**Sub questions:**

- *Where do you get your information from?*
- *What do you think of the information you hear and/or read?*
- *Are there issues or aspects of the COVID-19 outbreak which you would like to read and/or hear more about? (If yes, which ones?)*

## QUESTION 2 (10 MINUTES)

Aim: To gather information on the degree to which the participants are concerned about the COVID-19 pandemic. These answers also help contextualise the following responses.

**The moderator says:**
*Are you worried about the COVID-19 pandemic?*

**Sub questions**

- *Why are you (not) worried?*
- *What are you worried about?*
- *Who are worried about?*

## QUESTION 3 (10 MINUTES)

Aim: To gather the participants' experiences concerning their role prior to the COVID-19 pandemic.

**The moderator says:**
*To which extent do you think you were personally prepared for the COVID-19 pandemic?*

**Sub questions:**

- *What had you done within your home to prepare for such a pandemic?*
- *What had you done at work to prepare for such a pandemic?*
- *What had you done within your other social environments to prepare for such a pandemic?*

## QUESTION 4 (10 MINUTES)

Aim: To gather the participants' experiences concerning their role during the COVID-19 pandemic.

**The moderator says:**
*What are you personally doing now to reduce the impact of the COVID-19 pandemic?*

**Sub questions:**

- *What are you doing within your home to reduce the impact of the COVID-19 pandemic?*
- *What are you doing at work to reduce the impact of the COVID-19 pandemic?*
- *What are you doing within your other social environments to reduce the impact of the COVID-19 pandemic?*

## QUESTION 5 (10 MINUTES)

Aim: To gather the participants' expectations concerning their role when recovering from the COVID-19 pandemic.

**The moderator says:**
*How do you intend to help your community recover from the COVID-19 pandemic?*

**Sub questions:**

- *What do you intend to do you in your home to help the community recover from the effects of the COVID-19 pandemic?*
- *What do you intend to do at work to help the community recover from the effects of the COVID-19 pandemic?*
- *What do you intend to do in your other social environments to help the community recover from the effects of the COVID-19 pandemic?*

## QUESTION 6 (10 MINUTES)

Aim: To gather information on the participants' thoughts and opinions concerning the role of the ordinary citizen in the decision-making process during the COVID-19 pandemic.

**The moderator says:**
*Most of the decisions taken during the COVID-19 pandemic have a significant influence on everyone in society. Do you feel the need to express your opinions about the decisions that have been taken.*

**Sub questions:**

- **If so:**
    - *How have expressed these opinions// how would you like to express these opinions?*
    - *To whom would you like to express these opinions// to whom have you expressed these opinions?*
- *Do you think that those taking the decision regarding COVID-19 listen to your wishes and needs?*
    - *Why do you think that?*
    - *What could be better?*

## TABLE: INDIVIDUALS, GROUPS AND ORGANISATIONS INVOLVED IN THE COVID-19 OUTBREAK (10 minutes)

The moderator will ask the participants to fill in a table with actors and organisations that they believe are involved in the **preparedness** and **response** of the COVID-19 pandemic. The assistant will take notes of noteworthy comments and questions.

**The moderator says:**
*We all know that different people, groups and organisations are involved in the preparedness for pandemics such as the COVID-19 pandemic, as well as the response to the actual pandemic. We now have a short activity about the people, groups and organisations. You have received a document with a table, like the one I am now sharing with you* [**share screen**] *(see Appendix 2). In the column on the right you can write down all the individuals, groups and organisations that you think are involved in preparing for, and responding to the COVID-19 pandemic. In the left column you can write down the corresponding activities. If you know an actor is involved but you are not sure what its role is, just write down the actor's name in the column on the left. If you know an activity is being done but you are not sure who does this activity, just write down the activity in the column on the right. It really does not matter if you don't know many actors or activities, just please try to write down as many as you know.*

*You have 10 minutes to do this exercise on your own. Are there any questions? You can start.*

***[10 min]***

## QUESTION 7 (10 MINUTES)

Aim: To gather information on the participants' perception of the actors and tasks involved in the preparedness and response of the COVID-19 pandemic.

**The moderator says:**
*Thank you for filling in the table. Please take a picture of your table or save it as it would be great if you could send it to us after this session. When you look at your table, is it clear for yourself who does what concerning the COVID-19 pandemic?*

**Sub questions**

- *Is it clear which individuals, groups and organisations are involved in the preparedness of the COVID-19 pandemic?*
  - *If yes, what has made it clear for you?*
  - *If no, what would be necessary to make it clear?*
- *Is it clear which individuals, groups and organisations are involved in the response of the COVID-19 pandemic?*
  - *If yes, what has made it clear for you?*
  - *If no, what would be necessary to make it clear?*

**SHORT SUMMARY BY THE MODERATOR**

**BREAK (10 MINUTEN)**

## QUESTION 8 (10 MINUTES)

Aim: To gather information on the participants' opinions and thought on what the citizen's role should be during preparedness for an infectious disease outbreak such as COVID-19.

**The moderator says:**
*What do you think should be the citizen's role during preparation for an infectious disease outbreak such as COVID-19? [In other words: What do you think should be your role during preparation for an infectious disease outbreak such as COVID-19?]*

**Sub questions:**

- *What should be the citizen's role in preparing his or herself in his or her home for such a pandemic?*
- *What should be the citizen's role in preparing his or herself in the workplace for such a pandemic?*
- *What should be the citizen's role in preparing his or herself in other social environments for such a pandemic?*
- *Why should the citizen have this role?*
- *What is necessary to fulfil those roles?*
- *Who should the citizen work with to fulfil those roles? / Which organisation should the citizen work with to fulfil those roles?*

## QUESTION 9 (10 MINUTES)

Aim: To gather information on the participants' opinions and thought on what the citizen's role should be during response to an infectious disease outbreak such as COVID-19.

**The moderator says:**
*What do you think should be the citizen's role during response to an infectious disease outbreak such as COVID-19 once it is present? [In other words: What do you think should be your role during response to an infectious disease outbreak such as COVID-19?]*

**Sub questions:**

- *What should be the citizen's role within his or her home during the response to reduce the impact of COVID-19?*
- *What should be the citizen's role within his or her workplace during the response to reduce the impact of COVID-19?*
- *What should be the citizen's role within his or her other social environments during the response to reduce the impact of COVID-19?*
- *Why should the citizen have this role?*
- *What is necessary to fulfil those roles?*
- *Who should the citizen work with to fulfil those roles? Which organisation should the citizen work with to fulfil those roles?*

## QUESTION 10 (10 MINUTES)

Aim: To gather information on the participants' opinions and thoughts on what the citizen's role should be when recovering from a pandemic such as COVID-19.

**The moderator says:**
*What do you think should be the citizen's role when recovering from an infectious disease outbreak such as COVID-19 once it seems to be over? [In other words: What do you think should be your role during recovery after an infectious disease outbreak such as COVID-19?]*

**Sub questions:**
- *What should be the citizen's role within his or her home when the community is recovering from the COVID-19 pandemic?*
- *What should be the citizen's role within his or her workplace when the community is recovering from the COVID-19 pandemic?*
- *What should be the citizen's role within his or her other social environments when the community is recovering from the COVID-19 pandemic?*
- *Why should the citizen have this role?*
- *What is necessary to fulfil those roles?*
- *Who should the citizen work with to fulfil those roles? Which organisation should the citizen work with to fulfil those roles?*

## CONLCUSION (3 MINUTES)

The moderator summarises the discussion and asks if this a correct summary of what has been said. The moderator also asks if there are any final questions, remarks or issues to be raised.

The moderator thanks the participant for the presence and their valuable contribution. (S)he provides the participants with the instructions on where to send a picture or a scan of their tables to. (S)He reminds the participants that they will receive an electronic gift card.

The moderator wishes the participants a good day.

# Appendix 1 The moderator and the assistant's tasks

The moderator's tasks:

- Listen carefully, ask for clarification and interpret
- Try to ask the necessary questions that allow to concretise what participants say
- Ensure that the participants stay on topic (in the event a participant wishes to speak on a topic that is out of the scope, acknowledge the idea but also park it for later in the break or after the session)
- Encourage a dynamic discussion
- Ensure that there is flow and dynamism in the discussion
- Ensure that all participants have the opportunity to speak
- Ensure that the discussion is not lead by 1 or 2 individuals
- Give the participants room to discuss amongst themselves and ask question while ensuring that the 8 key questions are asked
- Accept silences and wait for participants to react to each other; the five second pause technique can be an appropriate response after a participant has said something as it invites participants other than the moderator to respond
- In case the participants don't understand a question, repeat it in an alternative form without providing them with possible answers
- Try to ask open-ended questions

The assistant's tasks:

- Take general notes on the content of the discussion, hoe the responds react to each other and the general atmosphere
- Keep track of time and prompting the moderator to move on when necessary
- Help prompt the moderator in case some responses remain vague or if some respondents are speaking significantly more than others
- Help the moderator in case they need assistance in leading the discussion

## Appendix 2 Table: Individuals, groups and organisations involved in COVID-19 outbreak

| Individuals, groups and organisations | Tasks |
| --- | --- |
| | |
| | |
| | |
| | |
| | |
| | |
| | |
| | |
| | |
