# Supplementary material for: The citizen’s perception of a shared responsibility during the COVID-19 management: Insights from a focus group study across four European countries
Source: PLoS One. 2025 May 27;20(5):e0322019. doi: 10.1371/journal.pone.0322019 (PMC12112160; doi:10.1371/journal.pone.0322019)
Supplement: S2 Annex — (PDF) [file pone.0322019.s002.pdf]

METC Utrecht
RIVM
LCI
To the attention of S. Kamga
Per e-mail: Sandra.kengne.kamga.mobou@rivm.nl

Our reference number MvdL/mb/21/500203
Date 18 February 2021
Subject METC- protocol number 21-111/C
Non-WMO research statement

Medical Research Ethics Committee

Contact:
Department of Research Review
Tel 088 75 563 76
(Morning Mon – Thursday)
info@metcutrecht.nl

Dear Ms. Kamga,

The Medical Research Ethics Committee (METC) has received the documents accompanying the research proposal number 21/111, titled "Understanding the citizen's role during COVID-19 preparedness, response and recovery" as submitted by Dr. A. Timen, and has considered whether an official approval of this study considering the Medical Research Involving Human Subjects Act (WMO) is required.

On the basis of the documents shown below, the MREC Utrecht Board concluded on 16 February 2021 that the WMO does not apply, based on one or both of the following reasons:

- It is not medical-scientific research as defined by the CCMO. The definition can be found on the CCMO's website: Uw onderzoek: WMO-plichtig of niet? | Onderzoekers | Centrale Commissie Mensgebonden Onderzoek (ccmo.nl)

  and/or

- Participants are not required to perform any action or behave in predetermined manners, for which approval is required.

This means that the METC has no legal task in the assessment of the content of this proposal. The commission would like to bring to your attention that it has only considered whether WMO applies, and this statement is not a permission to perform the study. You are responsible for conducting the study according to existing laws such as, but not limited to, the AVG (General Data Protection Regulation), the WGBO, and the policy of the institute within which the study will be conducted. There is no need to inform the METC about the study progress or finalization.

The commission considered the following documents:

Topic	Date received
A1. Accompanying email dated 09-02-2021	9 February 2021
A1. Non-WMO Form dated 09-02-2021	9 February 2021
C1. Research protocol SHARP WP6 Task 6.2.2. EN version 3 dated 09-02-2021	9 February 2021

It is recommended to discuss study changes and/or amendments that could be WMO relevant with the MREC.

Yours sincerely,

on behalf of the Medical Research Ethics Committee

Department of Research Review

This letter is sent without a signature, as it does not legally require one..

Copy

- Principal investigator: Dr. A. Timen, per e-mail: aura.timen@rivm.nl

To whom it may concern,
Referring to our letter of 18 February 2021 (reference number MvdL/mb/21/500203) it is confirmed that the Medical Research Involving Human Subjects Act (WMO) does not apply to the above mentioned study and that therefore an official approval of this study by the MREC Utrecht is not required under the WMO.

Your sincerely,
on behalf of the Medical Research Ethics Committee,

Department of Research Review

Our reference number MvdL/mb/21/500203
Pages 2/2
