# Supplementary material for: The citizen’s perception of a shared responsibility during the COVID-19 management: Insights from a focus group study across four European countries
Source: PLoS One. 2025 May 27;20(5):e0322019. doi: 10.1371/journal.pone.0322019 (PMC12112160; doi:10.1371/journal.pone.0322019)
Supplement: Attachment — Submitted filename: 20241203 Author response Plos One.docx [file pone.0322019.s005.docx]

Dear Prof. Schicktanz,

We would like to thank you and the reviewers once again for providing us with feedback to improve the quality of our manuscript, entitled “The perceptions amongst European citizens of shared responsibility during the COVID-19 management: A focus group study across four European countries”.

The research team has carefully considered and discussed the new comments. Below, you will find our point-by-point responses to all comments, and corresponding changes in the manuscript and where to find them. All significant changes have been marked. All authors have seen and approved the revised manuscript.

We hope this revised manuscript can be accepted for publication in PLOS ONE.

Yours sincerely,

Sandra Kengne Kamga

On behalf of the all the co-authors

**Additional Editor Comments:**

Dear authors,

we appreciate your revision efforts, but reviewer 1 has some concerns which I also share. Itthat you describe your study design according to main standards of qualitative focus group research, including giving detailed information about socio-demographics (incl. education, gender, profession, perhaps Covid-infection: affected vs. non.affectedness). Also problematic generalizations as outcome of a qualitative study should be avoided. Please replace Euroipean by naming only te countries you have examined (in the title, abstract, introduction, result and discussion parts). Discuss then more closely your findings for the respectives countries in comparison to other existing qualitative and quantitative studies of these countries (Trust-related issues into health sciences, governance, ). You can also discuss your results of your 4 countries with studies that have been undertaken in other countries but please make sure you compare apples with apples...

As qualitative studies help to buil hypothesis but not generalized sztatement, I also recommend that you apply a more reflective discussion style and develop rather an outlook part, how to test your hypothesis in future studies.

We are looking forward to recieve a revised vrsion of your manuscript

Sincerely, Yours Silke Schicktanz


Thank you for your comments. We have replaced “European” with the names of the four countries included throughout the manuscript. We have also referred to previous relevant literature from the four countries when discussing our results more frequently. Moreover, we have changed the wording of some sentences in the discussion to avoid possible overgeneralizations and we have provided suggestions for future research based on our results.

Unfortunately, we do not have more detailed socio-demographics than what has been provided here. We have, however, now reflected on this in the discussion.

Reviewers' comments:

Reviewer's Responses to Questions

**Comments to the Author**

1. If the authors have adequately addressed your comments raised in a previous round of review and you feel that this manuscript is now acceptable for publication, you may indicate that here to bypass the “Comments to the Author” section, enter your conflict of interest statement in the “Confidential to Editor” section, and submit your "Accept" recommendation.

Reviewer #1: All comments have been addressed

Reviewer #2: (No Response)

2. Is the manuscript technically sound, and do the data support the conclusions?

The manuscript must describe a technically sound piece of scientific research with data that supports the conclusions. Experiments must have been conducted rigorously, with appropriate controls, replication, and sample sizes. The conclusions must be drawn appropriately based on the data presented.

Reviewer #1: Yes

Reviseer #2: Partly

3. Has the statistical analysis been performed appropriately and rigorously?

Reviewer #1: Yes

Reviewer #2: N/A

4. Have the authors made all data underlying the findings in their manuscript fully available?

The [PLOS Data policy](http://track.editorialmanager.com/CL0/http:%2F%2Fwww.plosone.org%2Fstatic%2Fpolicies.action%23sharing/1/010f01932632c661-dc492e44-e272-48a6-a559-15ddf4cd3f42-000000/LB4lWyfcdrA5i8pPCdIWyxtKvNDCfobzMAIhwV0axIE=185) requires authors to make all data underlying the findings described in their manuscript fully available without restriction, with rare exception (please refer to the Data Availability Statement in the manuscript PDF file). The data should be provided as part of the manuscript or its supporting information, or deposited to a public repository. For example, in addition to summary statistics, the data points behind means, medians and variance measures should be available. If there are restrictions on publicly sharing data—e.g. participant privacy or use of data from a third party—those must be specified.

Reviewer #1: (No Response)

Reviewer #2: Yes

5. Is the manuscript presented in an intelligible fashion and written in standard English?

PLOS ONE does not copyedit accepted manuscripts, so the language in submitted articles must be clear, correct, and unambiguous. Any typographical or grammatical errors should be corrected at revision, so please note any specific errors here.

Reviewer #1: (No Response)

Reviewer #2: Yes

6. Review Comments to the Author

Please use the space provided to explain your answers to the questions above. You may also include additional comments for the author, including concerns about dual publication, research ethics, or publication ethics. (Please upload your review as an attachment if it exceeds 20,000 characters)

Reviewer #1: It seems to me that the authors have taken seriously my remarks and, according to their answer, the manuscript has improved sufficiently to be published.
I really appreciate the effort in providing context for each participant country phase of the pandemic. I also appreciate the details given on the methodology and coding process.

In sum, I think this is a great contribution, that has high value in terms of understanding a substantive question: what kind of expertise and legitimacy has the public in engaging with public-health policy decision-making? This paper analyses this problem in depth and is extremely informative.

I think it should be published.

Thank you for your compliments.

Reviewer #2: Thank you for addressing my comments and suggestions. However, I still have some concerns that I would like to share:
1. The vast amoung of literature on citizen perceptions on regulations and information during the COVID-19 pandemic is still not sufficiently covered in my view. As such, I find that the authors have not sufficiently addressed my first comment in the original review. Since the authors frame it as a European citizen perspective, they should not limit their literatur to the four countries investigated but take a broader view, at least on the European level. For example, publications that speak to the findings of this study and are able to contrast and refine the interpretation of them might be found in the German COSMO study (<https://projekte.uni-erfurt.de/cosmo2020/web/publications/>), the UK COSMO study (<https://cosmostudy.uk/publications>) or the pan-European SolPan project (<https://digigov.univie.ac.at/projects/solidarity-in-times-of-a-pandemic-solpan/solpan-publications/>). They report on citizen compliance, information behavior, trust in governments, etc. There is much more research published in addition to these three research initiatives from various European contexts and settings.

Thank you for your comments. We have now included more relevant literature on citizen perception in Finland, the Netherlands, Slovenia, and Spain in the Discussion section. Following the editor’s suggestion, we no longer frame the outcome as a European citizen perspective but focus on the four countries investigated.

2. I understand why the authors decided to stratify the focus groups on age, but the emphasis on the age gives the impression that the focuis groups reflect what people that age in that country generally think – which is not possible, given that 3-10 participants are hardly representative of a country’s age group. A more neutral framing of the focus groups (e.g., “Focus group 1, focus gropu 2”) might be helpful to avoid this flawed impression. (I do acknowledge that in some instances, the age matters, e.g. the criticism from young people that students were not sufficiently listened to. But I think it would be enough if the age group was mentioned in these rare occasions only).

We understand this concern. However, we have stated in the methods, that the aim of the recruitment is to hold an in-depth, explorative discussion with participants across different age categories, rather than to yield a representation sample of the population within the individual countries. We have again emphasized this in the strengths and limitations section.

3. Relatedly, I am missing some additional information on the study participants to estimate a little bit how well they represent the general citizen. Some demographic characteristics – e.g., how well participants were educated, whether they had children, whether they lived in a rural or urban setting – are highly influential on how they might speak about and value information and whether and how they trust politics.

Unfortunately, we do not have more detailed socio-demographics than what has been provided here. We have, however, now extensively reflected on this in the discussion.

4. The results present each focus group as a homogeneous entity with similar viewpoints and attitudes. Differences were only reported between age groups or between countries. Weren’t there any relevant discrepancies, conflicts, or differing viewpoints within the focus groups? And if not, why? Too homogeneous viewpoints would, again, raise my concern that the selection of participants is somewhat skewed towards a certain type of citizen. This is hardly avoidable in a qualitative study, but any biases in participant selection should be carefully analysed and reported.

The aim of this study was to identify key themes, and the differences identified were between the age groups and countries as has been documented. Although not all topics was not evoked or agreed upon by all individuals in the focus groups, we did not omit any explicit no oppositional opinions in our results. We have at times changed the wording of some sentences in the Results and Discussion section, to avoid potential overgeneralization. We have aimed to highlight that certain themes were discussed, rather than suggesting that all participants in particular focus groups had the same opinion. We also have now explicitly acknowledged a possible selection bias of participants in the strengths and limitations section of the Discussion. However, we also feel the probability of this bias has been minimized by the fact that the points of views were not homogenous across age groups or countries.

5. The aim of the study is, according to the authors, to “explore the European citizen’s perception of its role in the COVID-19 preparedness, response and recovery” also for future pandemic. However, the authors do not distinguish between preparedness, response and recovery in their results or discussion. This is problematic, since citizen participation might take different forms in times of acute crisis as opposed to more “normal” times.

Thank you for this remark. Participants were explicitly asked questions related to their roles during preparedness, response, and recovery. Although most of the (sub-) themes identified focused on the response phase, the manuscript also elaborates on some sub-themes specific for the preparedness and recovery phases, under the heading “the citizen’s actions during preparedness and response/recovery.”

6. Line 550: “Rather citizens seem to see a more passive role for themselves.” I find this statement problematic because it overly generalises and sees citizens as a homogeneous mass with similar interests and attitudes. It is also not a suitable interpretation of this study’s findings, in my view. If anything, the study shows how citizen perceptions on their own role in policymaking differ, with some wanting more active and others more passive roles. This should be made more nuanced, which is – in my view – the whole point in conducting qualitative studies like this one.

Thank you for your remark. We acknowledge this statement may have been an overgeneralization. This is the reason why we had already removed it in the previously revised manuscript. This statement is no longer in the most recent manuscript.

7. The results on trust and the observed country differences should be framed more cautiously, since the study participants might not represent the majority views in each country. The authors should find national polls on citizens’ trust in politics to back up their findings and clarify whether these are representative (e.g. whether they confirm that the Spanish and Slovenian populations are less trusting their governments than Dutch and Finnish populations).

We have now provided additional studies and national polls which back up our findings. These can be found in the Discussion.

I hope these comments will help the authors to improve their paper further. The methodology seems solid and is well explained and the rationale of the study is clear and relevant. But the relatively few participants from that many different countries poses a challenge to focus the results and discussion to speak meaningfully to the aim of the paper. I also think the authors could leverage more on their qualitative study design to add nuance to citizen perspectives rather than overly (and invalidly) generalise their findings.

Thank you for your comments. We hope the changes we have made to the manuscript have highlighted both the preliminary and relevant nature of our qualitative results.

7. PLOS authors have the option to publish the peer review history of their article ([what does this mean?](https://track.editorialmanager.com/CL0/https:%2F%2Fjournals.plos.org%2Fplosone%2Fs%2Feditorial-and-peer-review-process%23loc-peer-review-history/1/010f01932632c661-dc492e44-e272-48a6-a559-15ddf4cd3f42-000000/WF_TAShx-y_jXGu6GLEMmaWhBmrecVpD9MRlVh37E3o=185)). If published, this will include your full peer review and any attached files.


If you choose “no”, your identity will remain anonymous but your review may still be made public.


**Do you want your identity to be public for this peer review?** For information about this choice, including consent withdrawal, please see our [Privacy Policy](https://track.editorialmanager.com/CL0/https:%2F%2Fwww.plos.org%2Fprivacy-policy/1/010f01932632c661-dc492e44-e272-48a6-a559-15ddf4cd3f42-000000/aPJlcyqCtAXzNloFrIX6JcncT7c1ZQQJM8K1vGMMMwU=185).

Reviewer #1: **Yes:** David Rodríguez-Arias

Reviewer #2: **Yes:** Bettina M. Zimmermann

[NOTE: If reviewer comments were submitted as an attachment file, they will be attached to this email and accessible via the submission site. Please log into your account, locate the manuscript record, and check for the action link "View Attachments". If this link does not appear, there are no attachment files.]

While revising your submission, please upload your figure files to the Preflight Analysis and Conversion Engine (PACE) digital diagnostic tool, [https://pacev2.apexcovantage.com/](https://track.editorialmanager.com/CL0/https:%2F%2Fpacev2.apexcovantage.com%2F/1/010f01932632c661-dc492e44-e272-48a6-a559-15ddf4cd3f42-000000/kWwZHXM3xkKYTzI8rY8UPjw5pvQ6GLJbdQs0nCkrTYc=185). PACE helps ensure that figures meet PLOS requirements. To use PACE, you must first register as a user. Registration is free. Then, login and navigate to the UPLOAD tab, where you will find detailed instructions on how to use the tool. If you encounter any issues or have any questions when using PACE, please email PLOS at [figures@plos.org](mailto:figures@plos.org). Please note that Supporting Information files do not need this step
