# Supplementary material for: The citizen’s perception of a shared responsibility during the COVID-19 management: Insights from a focus group study across four European countries
Source: PLoS One. 2025 May 27;20(5):e0322019. doi: 10.1371/journal.pone.0322019 (PMC12112160; doi:10.1371/journal.pone.0322019)
Supplement: Attachment — Submitted filename: 20250313 Author response Plos One.docx [file pone.0322019.s006.docx]

Dear Ms. Howard,

We would like to thank you and the reviewers for reviewing our manuscript, entitled “The perceptions amongst European citizens of shared responsibility during the COVID-19 management: A focus group study across four European countries”.

We acknowledge the error that we had made in Figure 1 and have changed the figure accordingly. Furthermore, we have made minor changes in the references list, as we have added several hyperlinks to indicate where some of the references could be found. These changes have been marked in the manuscript.

We hope this revised manuscript can be accepted for publication in PLOS ONE.

Yours sincerely,

Sandra Kengne Kamga

On behalf of the all the co-authors
